# Cracking the cryptic code in amyotrophic lateral sclerosis and frontotemporal dementia: Towards therapeutic targets and biomarkers

**DOI:** 10.1002/ctm2.818

**Published:** 2022-05-12

**Authors:** Tetsuya Akiyama, Yuka Koike, Leonard Petrucelli, Aaron D. Gitler

**Affiliations:** ^1^ Department of Genetics Stanford University School of Medicine Stanford California USA; ^2^ Department of Neuroscience Mayo Clinic Jacksonville Florida USA; ^3^ Chan Zuckerberg Biohub San Francisco California USA

**Keywords:** ALS, FTD, TDP‐43, UNC13A

## Abstract

Amyotrophic lateral sclerosis (ALS) and frontotemporal dementia (FTD) are two devastating human neurodegenerative diseases. A hallmark pathological feature of both diseases is the depletion of the RNA‐binding protein TDP‐43 from the nucleus in the brain and spinal cord of patients. A major function of TDP‐43 is to repress the inclusion of cryptic exons during RNA splicing. When it becomes depleted from the nucleus in disease, this function is lost, and recently, several key cryptic splicing targets of TDP‐43 have emerged, including *STMN2*, *UNC13A*, and others. *UNC13A* is a major ALS/FTD risk gene, and the genetic variations that increase the risk for disease seem to do so by making the gene more susceptible to cryptic exon inclusion when TDP‐43 function is impaired. Here, we discuss the prospects and challenges of harnessing these cryptic splicing events as novel therapeutic targets and biomarkers. Deciphering this new cryptic code may be a touchstone for ALS and FTD diagnosis and treatment.

## 1 ALS AND FTD: TWO NEURODEGENERATIVE DISEASES WITH CLINICAL AND PATHOLOGICAL OVERLAP

Amyotrophic lateral sclerosis (ALS) is a fatal adult‐onset neurodegenerative disease characterised by selective motor neuron degeneration, which causes progressive muscle weakness, leading to paralysis and eventually death from respiratory failure.^1^ Up to 15% of ALS cases can be accompanied by cognitive, behavioural and language impairments consistent with a diagnosis of frontotemporal dementia (FTD).^2^ The discovery in 2006 of TDP‐43 pathology associated with both ALS and FTD placed ALS and FTD firmly on a spectrum, with similar underlying molecular mechanisms.^3^ TDP‐43 is an RNA‐binding protein encoded by the *TARDBP* gene that is normally localised in the nucleus. Nuclear depletion and cytoplasmic aggregation of TDP‐43 is a pathological hallmark in more than 97% of ALS cases and nearly 50% of cases with FTD (known as FTLD‐TDP).^1^, ^2^ In the nucleus, TDP‐43's normal function is to regulate several aspects of RNA processing, ranging from RNA transcription, alternative splicing and RNA transport.^4^

## 2 TDP‐43 IS A REPRESSOR OF CRYPTIC RNA SPLICING

**FIGURE 1:**
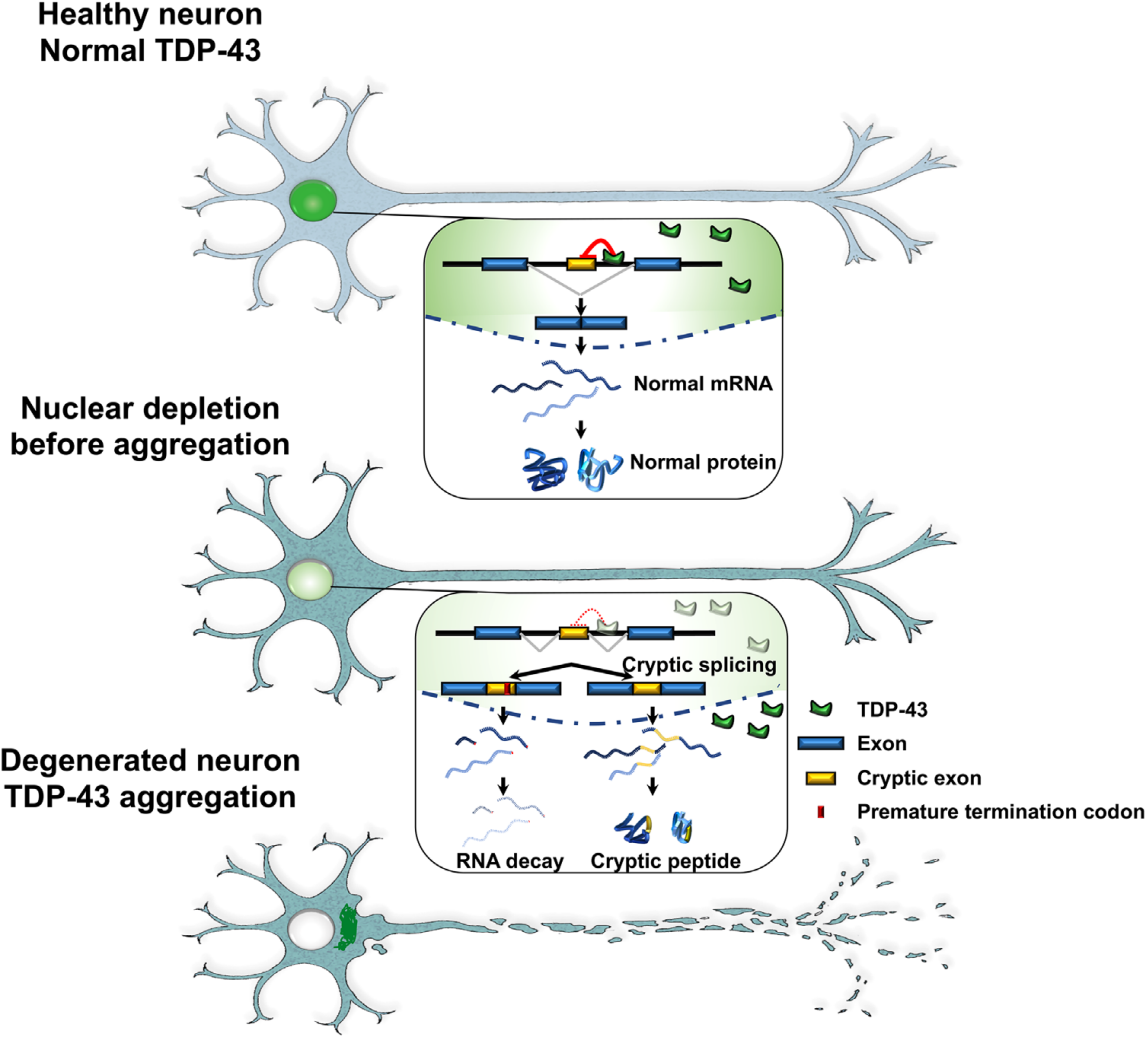
TDP‐43 is a repressor of cryptic RNA splicing. In healthy neurons, TDP‐43 localizes to the nucleus. Cryptic exons (yellow) are sequences located within introns that should not be included in the mature messenger RNA (mRNA) following splicing. As one of its major normal functions, nuclear TDP‐43 represses the inclusion of cryptic exons. In the early stages of disease, the depletion of TDP‐43 from the nucleus occurs before the appearance of cytoplasmic TDP‐43 aggregates. Nuclear TDP‐43 depletion leads to the inclusion of cryptic exons in mRNAs. These cryptic exons can destabilize the RNA, leading to its degradation or resulting in the production of aberrant peptides (cryptic peptides). In later stages of disease, TDP‐43 disappears from the nucleus and forms aggregates in the cytoplasm. Thus, both the loss of nuclear TDP‐43 and its cytoplasmic aggregation are associated with neurodegeneration in amyotrophic lateral sclerosis/frontotemporal dementia (ALS/FTD)

Among all these diverse roles, a major regulatory function of TDP‐43 has recently emerged—as a negative regulator of cryptic exons during splicing.^5^, ^6^ During normal gene splicing, exons, the parts of genes that code for proteins, are spliced together, and the intervening sequences, called introns, are spliced out to produce messenger RNA (mRNA), which contains the instructions to produce a protein product. However, some genes harbour sequences within introns that somehow resemble exons and can sometimes be mistakenly included in the mRNA (Figure 1). This can cause problems for a few reasons. These so‐called “cryptic exons” can disrupt the reading frame of the protein; they can destabilise the mRNA, leading to its degradation, or they can code for aberrant peptides that might cause problems if produced. TDP‐43 plays an important role in preventing these cryptic exons from sneaking into mRNAs. There are often binding sites for TDP‐43 beside these cryptic exon sequences, and when cells have functional TDP‐43, the cryptic exons are kept out of mRNA.^6^ However, when TDP‐43 function is impaired or it is depleted from the nucleus, these cryptic exons creep into the mRNAs of many different genes. This discovery that TDP‐43 represses cryptic exons offers a number of exciting possibilities for understanding the molecular underpinnings of ALS and FTD, leading to the development of sensitive new biomarkers and therapeutic targets. However, which cryptic splicing events are key for disease? Is there one main event or are many others important? Could there be different patterns of cryptic exon inclusion in different ALS and FTD subtypes and, if so, could these be harnessed as biomarkers?

## 3 STATHMIN 2 IS A KEY TDP‐43 CRYPTIC SPLICING TARGET IN HUMAN NEURONS

A breakthrough came in 2019 when two teams discovered an important human cryptic splicing target of TDP‐43.^7^, ^8^ They analysed RNA sequencing data from human neuronal cells and human neurons that had TDP‐43 depleted or harboured pathogenic TDP‐43 mutations. As expected, they found that impairing TDP‐43 function leads to changes in the expression of hundreds of genes. Among these, one in particular caught their attention—*STMN2*, which encodes stathmin 2, a protein that regulates microtubule stability in neurons. It was one of the most downregulated genes in their data sets. They quickly realised that the *STMN2* gene harbours a cryptic exon (exon 2a) that is normally not included in mature *STMN2* mRNA. The first intron of *STMN2* contains a TDP‐43 binding site, suggesting that TDP‐43 normally functions to repress inclusion of this cryptic exon. When TDP‐43 is lost or its function is impaired, exon 2a is incorporated into mature mRNA. Unfortunately, this exon harbours a stop codon and a premature polyadenylation signal (signals to tell the cell to stop producing the protein and RNA, respectively), which results in truncated *STMN2* mRNA and protein. Aberrant splicing and reduced STMN2 protein levels seem to be a major feature of familial and sporadic ALS cases (except those with *SOD1* or *FUS* mutations).^9^ This cryptic exon inclusion event in stathmin 2 appears to represent more than merely a readout of TDP‐43 dysfunction and is critical for neuronal impairments caused by loss of TDP‐43 function because both teams found that upregulation of STMN2 is able to rescue axonal regeneration defects caused by TDP‐43 depletion in human iPSC‐derived neurons.^7^, ^8^ These results support the idea that TDP‐43‐dependent neurodegeneration acts, at least in part, via defects in regulation of *STMN2* splicing. *STMN2* cryptic exon inclusion also seems to play a role in FTD. Truncated *STMN2* RNA is elevated in postmortem brain tissue from FTLD‐TDP‐43 cases but not in controls or cases with progressive supranuclear palsy (PSP), a different type of neurodegenerative disease with tau pathology instead of TDP‐43 pathology.^9^ Moreover, truncated *STMN2* levels correlate with the levels of phosphorylated TDP‐43 protein and with an earlier age of FTD symptom onset.^9^ It is intriguing to speculate that *STMN2* cryptic exon inclusion may even herald TDP‐43 inclusion formation, being present in a subset of the most vulnerable neurons even in the absence of TDP‐43 nuclear depletion or cytoplasmic aggregation. This point is important because these TDP‐43‐dependent cryptic exon splicing events in ALS and FTD might now allow the field to be able to detect the earliest events of TDP‐43 pathology and will suggest not only disease mechanisms but also novel therapeutic targets. The discovery of *STMN2* cryptic exon splicing in ALS and FTD shines a bright light on one key mRNA target. However, could there be more out there beyond *STMN2*? If so, it will lead to many new pathways for defining disease mechanisms and innovating sensitive biomarkers and therapeutic strategies.

## 4 IDENTIFICATION OF NOVEL TDP‐43‐REGULATED CRYPTIC SPLICING EVENTS IN FTD/ALS

Our group and another team recently identified dozens of novel cryptic exon splicing events regulated by TDP‐43 in human neurons, including at least one event that is directly connected to ALS and FTD by human genetics.^10^, ^11^, ^12^ Our group started with a tremendously valuable RNA sequencing data set generated by Dr. Edward Lee and colleagues.^13^ They wanted to identify changes associated with loss of TDP‐43 from the nucleus. They cleverly realised that they could use fluorescence‐activated cell sorting (FACS) to enrich neuronal nuclei that either contained TDP‐43 or did not and then perform RNA sequencing to compare the transcriptomes between TDP‐43+ and TDP‐43‐ neuronal nuclei from brains of FTD/ALS patients. They identified a multitude of interesting differentially expressed genes.^13^ However, we reasoned that we could reanalyse their data in a different way—not looking for differentially expressed genes as they did but instead searching for novel cryptic exon splicing events impacted by the loss of TDP‐43. We performed splicing analyses using new pipelines designed to detect novel splicing events^14^, ^15^ on RNA sequencing data from seven control samples (TDP‐43+ neurons) and seven samples containing TDP‐43‐negative nuclei sorted from patients. We identified 66 alternatively spliced genes with high confidence.^10^ We confirmed the *STMN2* cryptic exon and dozens of novel cryptic exon events. Perhaps most surprisingly, we found *UNC13A* to be one of the most significantly alternatively spliced genes. *UNC13A* immediately caught our attention because it is one of the top genome‐wide association study (GWAS) hits for ALS and FTD‐ALS.^16^, ^17^, ^18^, ^19^, ^20^, ^21^

In the absence of nuclear TDP‐43, a small cryptic exon is included in *UNC13A* mRNA, which introduces a premature stop codon, leading to a truncated protein. There are TDP‐43 binding sites in the intron harbouring this cryptic exon, suggesting that TDP‐43 directly regulates this splicing event. Indeed, depletion of TDP‐43 from human neuronal cells or motor neurons differentiated from induced pluripotent stem cells (iPSCs) caused inclusion of this cryptic exon in *UNC13A*. We also analysed a series of postmortem samples from the Mayo Clinic Brain Bank and from the New York Genome Center and found inclusion of this novel *UNC13A* cryptic exon in FTLD‐TDP patient brains and ALS cases.^10^ We showed that *UNC13A* cryptic exon expression levels are increased in the frontal cortices of patients with FTLD‐TDP, which is also positively correlated with phosphorylated TDP‐43 levels.^10^ At the same time, as in our studies, another team also identified cryptic exon inclusion in *UNC13A* as a consequence of TDP‐43 depletion.^11^ They did so by detailed analysis of RNA sequencing from iPS‐derived neurons depleted for TDP‐43. Thus, both teams have converged on cryptic splicing in the *UNC13A* gene as a consequence of TDP‐43 dysfunction.^10^, ^11^ This cryptic splicing event results in a decrease in *UNC13A* mRNA and protein, likely because the mRNA is degraded by a process of nonsense‐mediated decay (NMD; to maintain RNA quality, the cell has a way of detecting aberrant mRNAs that harbour premature termination codons) (Figure 1).^11^ *UNC13A* encodes a critical neuronal protein essential for communication between neurons—the release of neurotransmitter‐containing synaptic vesicles. It belongs to a family of genes originally discovered in worms because the animals that harboured mutations in these genes exhibited an uncoordinated phenotype due to deficits in neurotransmitter release.^22^ UNC13A is also essential for neuronal function in mammals because mice lacking this gene (also called Munc13‐1) die shortly after birth and have impairments in synaptic transmission.^23^ It is therefore likely that the cryptic exon inclusion event in *UNC13A* causes a loss of function, although we have not formally ruled out a role of potential cryptic peptides produced from this cryptic exon.

## 5 *UNC13A* CRYPTIC SPLICING EXPLAINS HOW IT FUNCTIONS AS A GENETIC RISK FACTOR FOR FTD/ALS

*UNC13A* is one of the top genetic risk factors for ALS and FTD,^24^ but how genetic variants (called SNPs, for single nucleotide polymorphisms) in *UNC13A* increase the risk for disease has remained unknown. Remarkably, it turns out that the top two risk SNPs in *UNC13A* are located in the cryptic exon containing intron with the top SNP located right in the cryptic exon itself! *UNC13A* is an enormous gene, and the SNPs could have been located anywhere; being located right where this cryptic exon is located argues strongly for a connection between the SNPs, risk for disease, and cryptic exon inclusion. Figuring out how GWAS hits connect to disease pathogenesis is a big challenge in general, but now we have a big bright light shining right on this cryptic exon splicing event and suggests testable hypotheses. Connecting genetics to pathology, both teams found that patient samples harbouring the risk alleles for these two GWAS SNPs have more *UNC13A* cryptic exon inclusion than FTLD‐TDP samples that do not contain the risk alleles. It does not seem that these SNPs are sufficient to cause cryptic exon splicing on their own because we did not detect them in the RNA sequencing data of healthy control samples. Instead, this event is likely TDP‐43‐loss dependent, and the GWAS SNPs might act like a sort of Achilles’ heel—lurking under the surface, not causing problems until TDP‐43 starts becoming dysfunctional. If so, there might be many more Achilles’ heels out there waiting to be discovered.

*UNC13A* cryptic exons are abundant in the brains of ALS and FTD patients, and the risk SNPs in the gene potentiate the accumulation of these cryptic exons.^10^, ^11^ However, does this affect disease outcomes? Indeed, stratifying FTD patients based on whether they had 0, 1 or 2 copies of the risk SNPs in *UNC13A* showed a strong association between the number of risk SNPs and a reduction in patient survival time after disease onset (patients with one copy of the risk SNPs live shorter than those with zero and those with two copies of the risk SNPs live even shorter),^10^ consistent with previous analyses indicating decreased survival in ALS and FTD patients harbouring *UNC13A* variants.^25^, ^26^, ^27^, ^28^ Thus, since genetic variants in *UNC13A* that increase cryptic exon inclusion are associated with decreased survival in patients, we strongly suspect that therapeutic strategies aimed at blocking this single splicing event could have a substantial therapeutic benefit (discussed below). ALS and FTD patients without the risk SNPs also have some cryptic splicing events (because they also have TDP‐43 pathology), so it is likely that therapeutic reduction of *UNC13A* cryptic splicing will be beneficial in these patients as well.

The discovery by both teams of a novel TDP‐43‐dependent cryptic splicing event in a bona fide FTD‐ALS risk gene now opens up many exciting new directions for validating *UNC13A* as a biomarker and therapeutic target in ALS and FTD (discussed below). However, we think this might be just the tip of the iceberg—we have dozens of novel TDP‐43 cryptic splicing events in hand, and we strongly suspect that there will be even more to discover. Are some of these also connected by human genetics to ALS and FTD? Can we decipher an FTD/ALS “code” comprised of specific patterns of cryptic exon splicing? In a broader sense, we now have a highly sensitive way of detecting the cellular consequences of TDP‐43 loss of function, even before frank TDP‐43 nuclear depletion and cytoplasmic aggregation. This will empower much more sensitive ways of visualising and studying ALS and FTD mechanisms and pathology in human patient samples.

## 6 CRYPTIC SPLICING EVENTS AS THERAPEUTIC TARGETS

How can we translate the discovery of TDP‐43‐dependent cryptic splicing targets into therapeutic strategies for ALS and FTD? Because TDP‐43 loss of function and gain of‐ function might both play a role in disease and given TDP‐43's essential role in cellular function,^29^ targeting the TDP‐43 protein itself is probably not warranted. However, what about the individual splicing targets? For the *UNC13A* cryptic exon inclusion event, which leads to decreased expression of UNC13A mRNA and protein,^10^, ^11^ one strategy is to restore normal UNC13A levels. This could be achieved using a gene therapy viral vector (e.g., adeno‐associated virus [AAV]) to deliver UNC13A. However, the challenges will be achieving the right level of UNC13A expression—too much might be deleterious—and in the right place.^30^ This type of approach seems to work for STMN2. Upregulating STMN2 levels with a lentivirus delivering the stathmin‐2 gene^7^ or by stabilising it via small molecule inhibition of c‐Jun N‐terminal kinase^8^ rescues impaired axonal regeneration and cell death caused by TDP‐43 loss in human‐induced pluripotent stem cell‐derived motor neurons. Moreover, it was recently shown that the introduction of the human *STMN2* gene is able to rescue the motor phenotype in mice deficient for *Stmn2* (*Stmn2‐/‐*).^31^

**FIGURE 2:**
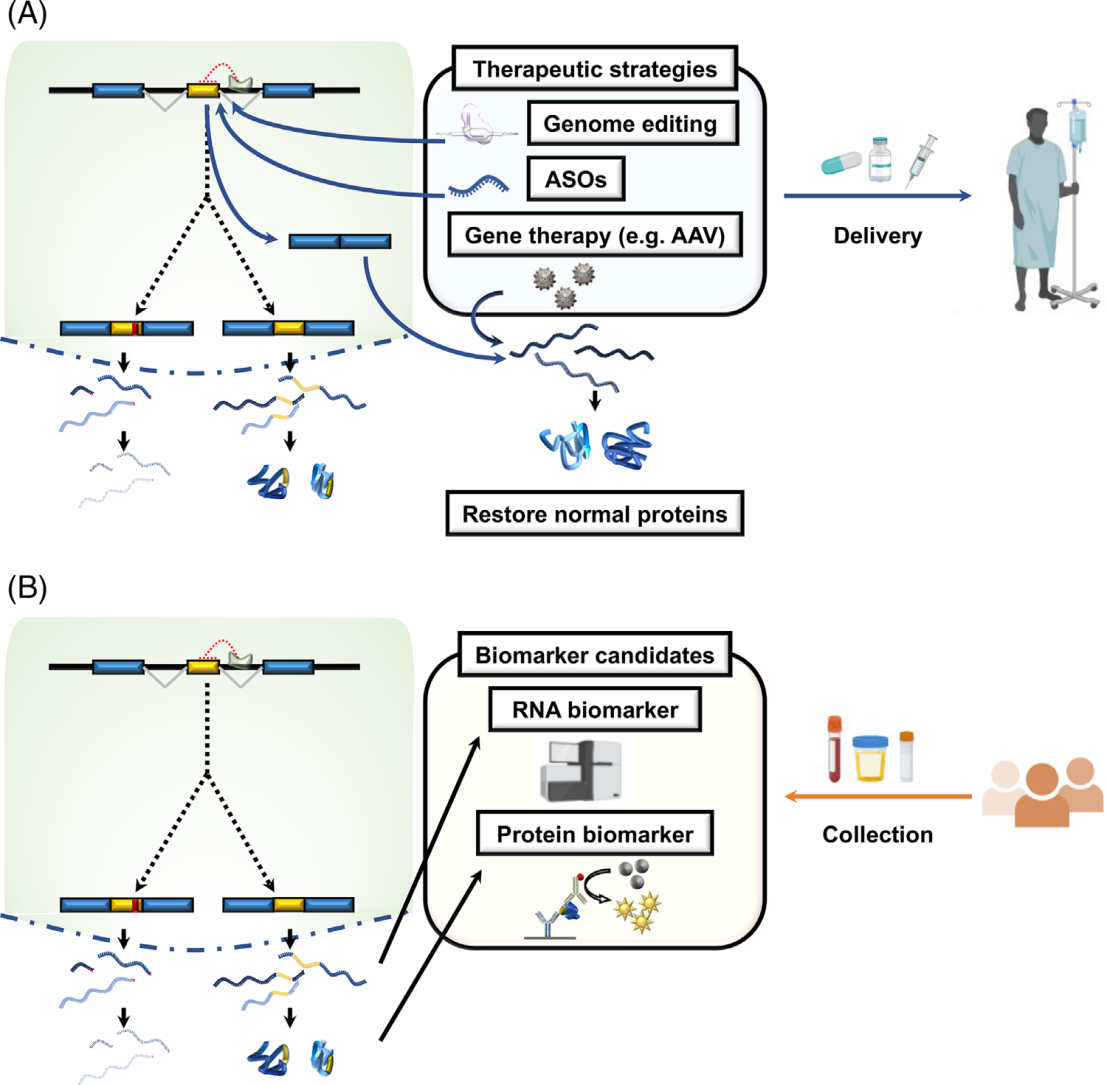
Cryptic splicing events as novel therapeutic targets and biomarker candidates. (A) Therapeutic strategies to prevent cryptic splicing events of key TDP‐43 targets could include CRISPR‐based genome editing of cryptic splice donor and acceptor sites, modified antisense oligonucleotides (ASOs) that trigger splice skipping, or gene therapy viral vectors (e.g., adeno‐associated virus [AAV]) to restore the expression of proteins that are downregulated by cryptic splicing events. (B) Cryptic splicing events can also serve as novel biomarker candidates, which could serve as sensitive readouts of TDP‐43 function. These biomarkers could be RNA‐based or protein‐based and could be detected in patient fluids (e.g., plasma or cerebrospinal fluid [CSF]). These biomarkers (or panels of multiple biomarkers) will aid in both early disease detection and the assessment of the efficacy of experimental therapeutics

Another approach could be to reduce or prevent cryptic splicing events from happening, even in the face of TDP‐43 dysfunction. Antisense oligonucleotides (ASOs) are chemically modified oligonucleotides that alter RNA targets, including triggering their degradation or modulating pre‐mRNA splicing.^32^ ASOs are emerging as a new generation of effective therapies for various neurodegenerative diseases, such as Duchenne muscular dystrophy, spinal muscular atrophy (SMA) and ALS.^33^ For individual cryptic splicing targets, such as *UNC13A* and *STMN2*, ASOs can be designed to specifically target and block the cryptic splicing sites on the pre‐mRNA. Indeed, the remarkable therapeutic effects of the splice‐skipping ASO nusinersen in SMA^34^ suggest the possibility of using a similar approach to correct untoward splicing events in ALS and FTD (Figure 2A). This ASO approach could be useful not only for preventing cryptic exon inclusion events caused by TDP‐43 loss of function but also for preventing “skiptic exon” events owing to TDP‐43 gain of function (i.e., exons that are inappropriately skipped because TDP‐43 binds nearby).^35^ A big question is now how many of these cryptic splicing events must be rescued to achieve therapeutic benefit. If it is only one or two (e.g., *STMN2* and *UNC13A*), then it seems feasible to design multiple ASOs targeting these regions. However, if many of the TDP‐43 splicing targets are important for disease, then other more global approaches will be needed to correct these events.

As genome editing approaches, including RNA editing, have become optimised for clinical application,^36^ a tantalising prospect is the ability to specifically edit cryptic splice acceptor sites, thereby rendering the cryptic exon impervious to TDP‐43 loss of function (Figure 2A). Genome editing still has several technical hurdles to overcome before clinical application, such as off‐target effects and delivery systems, but has the potential to be the most fundamental therapeutic approach for ALS/FTD.

## 7 CRYPTIC EXONS AS NOVEL BIOMARKER CANDIDATES

In addition to serving as therapeutic targets, these cryptic exon inclusion events can simultaneously be harnessed as biomarkers for diagnosis and prognosis and for evaluating the efficacy of experimental therapeutics.^11^ In ALS, several candidate fluid biomarkers have been reported, such as vascular endothelial growth factor (VEGF),^37^ neurofilament light chain protein (NfL)^38^ and urinary p75^ECD^.^39^ However, because these likely reflect neurodegeneration in an unspecific way, rather than being specific to ALS or FTD, none of them is definitive.^40^, ^41^ The next step is now to harness these newly discovered TDP‐43‐dependent cryptic splicing events and validate them in ALS and FTD patient tissue and biofluids (e.g., cerebrospinal fluid [CSF], blood urine) (Figure 2B). Because some of the cryptic exon splicing events also result in the production of cryptic proteins and peptides, it is highly likely that the levels of these proteins will reflect the extent of TDP‐43 dysfunction in the brain. In other words, monitoring the levels of cryptic proteins in patient samples could address one of the longest standing challenges in the ALS and FTD field: how to detect and monitor TDP‐43 pathobiological burden in vivo. A priori, it may not seem obvious that cryptic peptides could even be produced or play any role in pathology. However, as we have learned with another major genetic cause of ALS and FTD, mutations in the *C9ORF72* gene^42^, ^43^ and aberrant peptides produced from GGGGCC repeat expansion are abundant contributors to the pathology of c9FTD/ALS.^44^, ^45^, ^46^ We did not have the right tools to detect them. Now we do (powerful antibodies for detection by immunohistochemistry and sensitive ELISA‐based quantitative assays from biofluids)^47^, ^48^ and it has illuminated completely new facets of ALS and FTD pathology and has pointed the way to new disease mechanisms and therapeutic strategies.^49^, ^50^, ^51^, ^52^ Therefore, even though we cannot yet detect cryptic peptides predicted to be produced from cryptic splicing products, we strongly suspect that once the field develops the right tools, it will unveil completely new facets of ALS and FTD pathology. Stay tuned.

These new reagents will play an outsize role in detecting disease early, monitoring its progression and assessing whether candidate therapies are having an effect. Efforts should be launched to establish novel tools to detect cryptic proteins and peptides and to verify them in human samples. Then, with these in hand, we will need to evaluate the diagnostic efficacy of each marker for ALS and FTD and build prognostic biomarker panels. We envision a panel of high‐quality reagents to detect cryptic exon events in ALS and FTD biospecimens and then perform detailed clinical assessments. This will give the field an unprecedented lens through which to view disease—even in parts of the brain where TDP‐43 is still apparently in the nucleus but dysfunctional. It may give us a glimpse at the early events of pathogenesis and help stratify ALS and FTD patients for the design of more effective clinical trials.

There will be challenges along the road to cryptic exon biomarker development. It might be difficult to detect putative cryptic exon‐derived peptides (cryptic peptides). For example, even though the *STMN2* cryptic exon is highly abundant,^7^, ^8^, ^9^ thus far, detecting the cryptic peptide produced from *STMN2* has remained elusive. Other cryptic peptides may not be translated, owing to nonesense‐mediated mRNA decay (NMD), or may be cleared immediately and thus impossible to detect. Moreover, even if they are translated, they may be very low in quantity or may not be released outside the cell. The success of these biomarker efforts also hinges on the ability to produce high‐quality cryptic peptide‐specific antibodies. Methods are now available to sensitively detect changes in RNAs expressed in the brain by noninvasively analysing the blood^,^ ^53^ so in addition to developing protein‐based biomarker assays, those that detect the RNA products may also be suitable. Perhaps combining multiple biomarkers (e.g., *STMN2* and *UNC13A*) will provide more sensitivity and specificity than relying on single cryptic splicing events. If a cryptic peptide can be identified in serum or CSF, it might reflect the earliest signs of TDP‐43 dysfunction (even before cytoplasmic aggregation).^9^ It is axiomatic that the earlier we can detect disease, the better chance we will have to be successful in therapeutic intervention. We are learning these lessons with the development of promising therapies for SMA^54^ and from readouts of clinical trials targeting human ALS disease genes.^55^, ^56^

## 8 CONCLUDING REMARKS

The emergence of aberrant splicing events as a disease mechanism in ALS and FTD^7^, ^8^, ^10^, ^11^, ^57^ opens many exciting new directions for devising therapies and powerful new biomarkers. A challenge now will be to determine which of these dozens of new TDP‐43 splicing targets contribute to disease and which do not. If it is just one or a few targets, then developing therapies to tackle each of them will be feasible. However, if many of them contribute to disease simultaneously, it will be very difficult to target them one by one, and other approaches focused on improving TDP‐43 function^58^, ^59^ or restoring cryptic exon repression^60^ might be warranted. Because of the strong connections between *UNC13A*, cryptic exon inclusion and human genetics^10^, ^11^ and the dramatic effects of *STMN2* cryptic splicing on axon regeneration,^7^, ^8^ it seems that pursuing therapeutic strategies targeting *STMN2* and *UNC13A* is the right place to start. Meanwhile, efforts should be underway to identify additional candidate genes that may serve as therapeutic targets and biomarkers.

## CONFLICT OF INTEREST

Aaron D. Gitler is a scientific founder of Maze Therapeutics.
